# Internal consistency and construct validity assessment of a revised *Facts on Aging Quiz* for Flemish nursing students: an exploratory study

**DOI:** 10.1186/1471-2318-14-128

**Published:** 2014-12-03

**Authors:** Elisa Van der Elst, Mieke Deschodt, Melanie Welsch, Koen Milisen, Bernadette Dierckx de Casterlé

**Affiliations:** University Hospitals Leuven, Herestraat 49, 3000 Leuven, Belgium; Centre for Health Services and Nursing Research, Department of Public Health & Primary Care, KU Leuven, Kapucijnenvoer 35/4, 3000 Leuven, Belgium; Vzw Asster, Halmaalweg 2, 3800 Sint-Truiden, Belgium

**Keywords:** Education, Geriatric nursing, Knowledge, Nursing, Nursing students, Palmore’s Facts on Aging Quizzes, Psychometrics, Questionnaires, Validation studies

## Abstract

**Background:**

Since more people are reaching older and older ages, healthcare systems are becoming in need of more and more knowledgeable nurses to meet the specific health care needs of older persons. Several instruments exist to measure and evaluate students’ knowledge of older persons, ageing, and gerontological care; however, unequivocal evidence on their use and psychometric properties is scarce. The aim of the study was to validate a revised version of Palmore’s *Facts on Aging Quiz* (*FAQ*).

**Methods:**

A cross-sectional, exploratory study was conducted. Palmore’s *FAQ* version 1 and *Facts on Aging Mental Health Quiz* were used as bases for the development of a revised *FAQ* instrument. Three researchers translated these instruments into Dutch. A panel of nine experts in geriatric research and gerontological care evaluated the translation and the face and content validity of the instrument. We used a cross-sectional, exploratory design to assess its internal consistency and construct validity. Cronbach’s alpha coefficients, exploratory factor analysis, and the known-groups technique were used for these analyses.

**Results:**

Based on the experts’ consensus, a revised version of the *FAQ*, consisting of 36 items, was produced. Exploratory factor analysis did not reveal underlying constructs suggesting that the revised version encloses a more general concept of knowledge (e.g. about older persons, aging, gerontological care). Using the known-groups technique, we validated the instrument, showing that it discriminates between the knowledge of first- and third-year nursing students. The overall Cronbach’s coefficient of 0.723 was acceptable and changed minimally (from 0.708 to 0.724) when items were removed.

**Conclusion:**

We conclude that the revised version of the *FAQ* can be used to properly evaluate nursing students’ knowledge about older persons and gerontological care, as reasonable reliability and validity were established for this revised version of the *FAQ*.

**Electronic supplementary material:**

The online version of this article (doi:10.1186/1471-2318-14-128) contains supplementary material, which is available to authorized users.

## Background

Society is undergoing major demographic changes. People are reaching older ages, and as life expectancy increases, the proportion of persons older than 65 increases. It is expected that the number of very old (85 and over) persons will especially continue to increase and occupy an ever-increasing proportion of the overall population [1]. This ageing of the population has a direct impact on healthcare, since older persons tend to have disproportionately more chronic illnesses and disabilities. Moreover, as a lot of these older persons will remain at home longer, available access to non-medical and medical services in the community will be essential [2–4]. In general, a multidisciplinary team approach using gerontology and geriatric expertise is recommended in order to provide high-quality care to the ageing population [5, 6]. To provide high-quality care, future healthcare systems are increasingly in need of more knowledgeable nurses in order to meet the specific healthcare needs of older people [5–7]. Nurses are the primary caregivers for many older persons, and because of the uniqueness of their profession, their role is of great importance in designing, implementing and evaluating health care of older adults in primary, hospital, and long-term care [8–10]. It is often the nurse who has close and intense interactions with the older care recipient [11, 12]. In particular, they play an important role in the early detection and diagnosis of specific nursing problems, for example, bedsores, delirium, and fall risk [13–15]. Because older people in healthcare settings often present with a frailty profile, limited homeostasis, active multipathology, comorbidity, multipharmacology, disturbed pharmaco-kinetics, and psychosocial problems, they are in need of a different approach than adult patients with regard to the detection of healthcare problems and the maintenance and promotion of their current health care status [16, 17]. Nurses, and healthcare professionals in general, for example, often misperceive changes in cognitive and functional status as a normal consequence of aging, which may result (in)directly in negative outcomes, such as an accelerated deterioration of overall health [18]. Therefore, the health care of older patients should extend beyond the traditional medical management of illness and requires evaluation of multiple issues including physical, cognitive, affective, social and environmental components that influence an older adult’s health [17]. Although their role in providing care to the older patient is critical, studies show that some nurses and nursing students lack adequate knowledge about ageing, and do not possess the competencies or attitudes necessary for providing quality care to older persons [19–22].

The American Association of Colleges of Nursing (AACN) in collaboration with The John A. Hartford Foundation (2000), composed a document titled ‘Older adults: recommended baccalaureate competencies and curricular guidelines for geriatric nursing care’ that provides a description of the basic nursing competencies needed to guarantee high-quality care for older people, aiming to help educators implement specific gerontology content into their baccalaureate nursing education programmes [23]. However, although many nursing education programmes have integrated geriatric nursing into their curriculum [24, 25], the majority of nurses and nursing students also have inadequate experience in geriatric care and a rather negative attitude towards the care of older persons [26–29].

Taking into account all of the above, a valid and reliable assessment tool is needed to determine the state of gerontological knowledge nurses possess and to assess what they know about geriatric aspects of caring for older patients. Many tools have been developed for this purpose. A comprehensive literature review revealed an extensive body of literature about nursing and medical students’ knowledge of older persons and ageing [21, 22, 30, 31]. There are different measurement instruments that have been used, such as *Palmore’s Facts on Aging Quizzes (FAQs)* [32–35]; the *Geriatric Knowledge Self-Assessment Tool* [21]; the *Alzheimer’s Disease Knowledge Test* [30, 36]; the *UCLA Geriatrics Knowledge Test* [37, 38]; and the *University of Michigan Geriatrics Knowledge Test* [39, 40]. Most of these instruments are designed to measure medical students’ knowledge of older persons and ageing, and are therefore more focused on the medical aspects of ageing [37, 39]. Other instruments are rarely used now, and data on their psychometric properties are meagre [21, 30]. The most widely used instruments to measure nursing students’ knowledge are the *FAQs: Facts on Aging Quiz version 1 and 2* (*FAQ1* and *FAQ2*) and *Facts on Aging Mental Health Quiz (FAMHQ)* [32–34].

The *Palmore’s FAQs* were originally developed to encourage group discussion among healthcare workers about older persons and their care. Palmore’s quizzes have been described as being valid (e.g., face and known-groups validity) and reliable (e.g., internal consistency) when used in various groups of learners [27, 32–34]. Laner stated that *FAQ1* measures knowledge and factual learning independently from the specific content of courses being taught [35]. The *FAQ1,* as well as the *FAMHQ,* originally consisted of 25 items in ‘true’ or ‘false’ response format. *FAQ1* is designed to measure knowledge about basic physical, mental, and social factors of ageing; *FAMHQ* focuses more on assessing knowledge about the cognitive and mental aspects of older persons and ageing. Total scores of both instruments can range from 0 to 25, with higher scores reflecting greater knowledge about ageing and mental health of older persons [32–34].

Despite different publications and statements concerning the *Palmore’s FAQs* being valid and reliable, available evidence supporting these notions is still poor, and discordance appears to persist among researchers [26, 41, 42]. Several studies have re-evaluated the different versions of the FAQs and report a low internal consistency with Cronbach’s alphas varying from 0.45 to 0.66 [43–46]. Also, the study of Miller et al. revealed a major shortcoming of the *FAQ1*, as most items have an underlying negative bias [41]. If the items were worded in a positive way, the respondents might have higher scores, indicating richer, more complete knowledge about ageing and gerontological care.

There also appear to be problems for the alternate versions of *Palmore’s FAQs.* Seufert et al. [26] and Obiekwe [44] compared the different versions of the *FAQ* (1 & 2), and concluded these are not actually alternate measures, despite Palmore’s earlier assertions [33]. Furthermore, Lusk et al. [43] and Norris et al. [42] tried to perform factor analysis on *FAQ1*, and both found unstable factor structures, many substantively incoherent and different factors, and overall low factor loadings.

The situation is similar for the *FAMHQ*. Actually, the literature on the psychometric properties of the *FAMHQ* is remarkably scarce, if not completely obscure.

### Aim

Motivated by the above shortcomings of the *Palmore FAQs*, the purposes of the present study are (1) to develop a revised version of the instrument that addresses these deficiencies; and (2) to assess the psychometric properties of this revised version, specifically, its reliability and validity.

## Methods

The *FAQ1* and *FAMHQ* were used as a basis for developing the revised FAQ [32–34]. Response categories for each item were ‘true’, ‘false,’ or ‘don’t know’ [47].

### Validation based on expert agreement

#### Phase 1: translation of the FAQ1 and FAMHQ

Both instruments (*FAQ1* and *FAMHQ*) were translated into Dutch (Additional files 1 and 2) and then adapted or revised by the researchers. Four nursing researchers were involved in this process: two with extensive expertise in care for older people (MD, KM), the other with extensive expertise in nursing education (BDdC), and a student in Master of Science in Nursing, with a specialization in geriatric care (MW). Adaptation consisted of retaining or removing items from the translated *FAQ1*, adding *de novo* items, and adding items to the *FAQ* that originally appeared in the *FAMHQ*. Adaptation was based on discussions with the external expert panel of educators, taking into account the different subjects (content), length, and user friendliness of the adapted *FAQ*. We call this adaptation the revised *FAQ* (Additional file 3).

#### Phase 2: expert agreement on content and face validity

Content and face validity were further assessed by an external expert panel of educators at Flemish institutions involved in gerontology/geriatric education. Evaluation of content and face validity was based on oral agreement and consensus from the different experts. Relevance, clarity, and adjustments of the different items were thoroughly discussed and evaluated by the researchers and the expert panel.

### Psychometric testing based on exploratory study

After the translation and adaptation phases, and after reaching a consensus about content and face validity of the revised instrument, we conducted an exploratory pilot study to further evaluate other psychometric properties of the instrument, i.e., reliability and construct validity.

#### Design

A cross-sectional design was used to evaluate the internal consistency and construct validity.

#### Sample/participants

The study population for this investigation consisted of all first- (n = 3565) and third-year (n = 2066) baccalaureate nursing students in colleges in Flanders, Belgium. In December 2009, all colleges that offered nursing education (n = 18), received information about the study’s research objectives and an invitation to collaborate. Each department or college that decided to cooperate was asked to assign a contact person.

#### Data collection

Data were collected using a convenience sample of nursing students and proceeded from February 2010 until April 2010. All participants were asked to fill out the revised *FAQ*. Besides completing the knowledge assessment, a number of demographic and educational variables were collected from each participant: age; gender; educational background (secondary school, college, or university degrees); and current educational programme enrolled in (first- or third-year student, internship experiences, chosen specialization [e.g., geriatric nursing, etc.]).

#### Ethical considerations

Ethical approval for multi-centred research was obtained from the Ethics Committee of the KU Leuven. Participation was voluntary and informed consent was assumed if the questionnaire was filled out. Anonymity and confidentiality were guaranteed, both at the college and student level. Before completing the questionnaire, oral and written information about the aims and relevance of the study was provided. All participants had the opportunity to discuss their thoughts or questions with the researcher or assigned contact person.

#### Data analysis

Exploratory factor analysis was performed on the tetrachoric correlation matrix for binary variables (i.e. true versus false/don’t know) to explore the underlying constructs of the items included in the adapted or revised *FAQ*. Using multivariate exploratory techniques, together with Oblique rotations and factor extraction (based on scree plots), eigenvalues (>1.4), and factor clarity, we made an attempt to compose an acceptable factor solution [48]. The model fit was evaluated calculating the incremental fit index (CFI > 0.95), Standardized Root Mean Square Residual (SRMR < 0.08) and Root mean square error of approximation (RMSEA < 0.06). In addition, the known-groups technique was applied to data from first- and third-year nursing students in order to determine the distinctive character of the adapted or revised *FAQ* for these two groups.

Cronbach’s alpha scores were calculated to evaluate the internal consistency of the instrument.

Statistical software (IBM SPSS Statistics version 17 and Mplus version 7.11) was used to analyse the data, with a predetermined significance level of p < 0.05.

## Results

### Validation based on experts’ agreement

The *FAQ1* and *FAMHQ* were translated into Dutch by four of the authors (MD, MW, KM & BDdC), who were native speakers of Dutch and who also had extensive knowledge of written and spoken English. When a decision was made either to retain or remove certain items from the new instrument, it was thoroughly discussed with the external expert panel of educators, taking into account the different subjects (content), length, and user friendliness of the revised *FAQ* (See Table 1).

**Table 1:** **Rotated component matrix with the revised and extended version of** ***FAQ*** **and** ***FAMHQ***

	Component						
Item	1	2	3	4	5	6	7
*(1) The majority of old people (age 65 or older) are senile (have defective memory, are disoriented, or demented).*	0.507						
(2) The majority of old people have no interest in, or capacity for, sexual relations.	0.474			0.342			
(5) The majority of old people feel miserable most of the time.	0.889						
(7) More than 25% of the aged are living in long-stay institutions (such as nursing homes, mental hospitals, homes for the aged, etc.).	0.326			0.246			
(8) Older workers usually cannot work as effectively as younger workers.	0.385	0.300					
(9) The majority of old people are unable to adapt to change.	0.489						
(10) The majority of the aged are healthy enough to do their normal activities without help.	0.353			0.256			
(13) In general, old people tend to be pretty much alike.	0.498						
(16) The majority of old people are socially isolated.	0.548				0.414		
(3) The five senses (sight, hearing, taste, touch, and smell) all weaken in old age.		0.264					
(4) Lung vital capacity declines in old age.		0.293					
(6) Physical strength declines in old age.		0.405					
(11) Old people usually take longer to learn something new.		0.486					
(14) Older people tend to react more slowly than younger people do.		0.671					
(22) The majority of old people say they are seldom irritated or angry.			0.519				
**(24) Life expectancy at age 65 is about the same for men as for women.**			0.146				
*(25) The majority of persons over 65 have some mental illness severe enough to impair their abilities.*			0.544				0.324
*(26) Cognitive impairment (memory loss, disorientation, or confusion) is an inevitable part of the aging process.*			0.388				
*(28) There is no treatment (that leads to a cure) for Alzheimer’s disease.*			0.429				
*(29) Most patients with Alzheimer’s disease act the same way.*			0.252				
*(30) It is best to avoid talking to demented patients, because it may increase their confusion.*			0.791				
*(31) Demented patients should not be allowed to talk about their past because it may depress them.*			0.749				
(17) Older workers have fewer accidents than younger workers do.				0.380			
(18) At present more than 30% of the population are 65 years or older in Flanders.				0.316			
(19) The majority of medical practitioners give low priority to working with or caring for the aged.				0.257			
(21) The majority of old people is working or would like to have some kind of work to do (including housework, volunteer work, care for grandchildren, etc.).				0.335			
(12) Depression is uncommon in older people.					-0.325		
(15) The majority of old people say they are seldom bored.					0.479		
*(27) Alzheimer’s disease is the most common type of chronic cognitive impairment among the aged.*					0.219		
*(32) The prevalence of cognitive impairment increases in old age.*						0.746	
*(33) The elderly have fewer sleep problems than younger persons.*			0.275			0.372	
*(34) Mental illnesses are more common (more prevalent) among the elderly with less income and education.*						0.311	
(20) The majority of old people have incomes below the poverty line (between €700 and €900 a month).							0.259
**(23) The risk of physical injury (e.g., broken hip or wrist) is the same in older persons as it is in younger persons.**					0.259		0.311
*(35) The majority of nursing home patients suffer from mental illness.*							0.737
*(36) Major depression is more prevalent among the elderly than among younger persons.*							0.447

Regular font = items originating from the Facts on Aging Quiz (FAQ) version 1.

Bold font = items were added (as recommended by the Flemish expert panel).

Italicized font = items originating from the Facts on Aging and Mental Health Quiz (FAMHQ).

Our revised instrument consisted of a mixture of items from the FAQ1, FAMHQ, and new items. Of the original 25 items of the *FAQ1,* 22 items were retained (See Table 1) and translated into Dutch. The two omitted items were: ‘*Aged drivers have fewer accidents per driver than those under the age of 65*’ and ‘*Older people tend to become more religious as they age*’. The expert panel judged these items irrelevant. A third item, ‘*The health and economic status of old people will be about the same or worse in the year 2010 (compared to younger people)*’ was excluded because this statement is dated. Following the recommendations of the Flemish expert panel, two items about life expectancy and risk of physical injury were added: ‘*The risk of physical injury (e.g., broken hip or wrist) is the same in older persons as it is in younger persons*’ and ‘*Life expectancy at age 65 is about the same for men as for women*’. At this point, then, the new assessment instrument comprised 24 items.

To the 24 items of the revised *FAQ1,* 12 items from the *FAMHQ* (See Table 1) were added. The other 13 items of the *FAMHQ* were not included for two reasons: (1) they were not considered to be relevant to the assessment of current nursing students’ knowledge of older persons and their care, or (2) they were too narrowly focused on knowledge about psychiatric care for older persons.

Thus, the first provisional version of the adapted *FAQ* comprised 36 items, 22 items from the original *FAQ1,* two new items about life expectancy and risk of physical injury, and 12 items from the original *FAMHQ* (See Table 1).

A group of nine experts (the expert panel) evaluated this first provisional version. After making some minor linguistic adjustments, all experts agreed on the face validity, user friendliness, relevance, and content of the items included in the revised 36-item version of *FAQ*.

### Psychometric testing based on a pilot study

#### Sample characteristics

Of the 18 colleges that offer nursing education in Flanders, 13 agreed to participate (response rate = 72.2%). This resulted in a sample of 1141 nursing students who completed the questionnaire, of whom 70.2% (n = 801) were first-year nursing students and 29.8% (n = 340) were third-year nursing students, respectively; representing 20.3% of the total number of first- and third-year nursing students in Flanders in the academic year 2009 – 2010. The majority was female (77.5%), with a mean age of 21.1 years (range = 18-53 y; SD = 4.987). Most students had graduated with a technical secondary education (50.8%) or a general secondary education (42.1%) and did not pursue any other form of higher education (70.8%), besides their current nursing programme. Of those who had any prior education, 18.9% (n = 60) gained some experience in care for older persons during this education. In general, 75.5% (n = 858) of the responding nursing students gained some experience in caring for older people before or during the nursing education programme they were pursuing at the time of the assessment for the present study. About a third (31.1%; n = 355) of the students had the opportunity to do an internship at a geriatric ward. A minority (4.6%; n = 53) completed an internship at a gerontopsychiatric ward.

#### Construct validity and internal consistency

We used exploratory factor analysis on the tetrachoric correlation matrix for binary variables to examine the underlying constructs of the items included in the adapted/revised *FAQ*, since previous research failed to reveal a distinct factor structure of the original *FAQ* [37, 39]. After attempting different factor solutions, we were unable to produce a simple structure with this method. Factor extraction based on retention of factors with eigenvalues >1.40, after Oblique rotation, resulted in a seven-factor solution. This model fitted the data well with a CFI of 0.983, SRMS of 0.042, and RMSEA of 0.010 (95% CI 0.00 – 0.015). Although most communalities were low among the factors (ranging from 0.146 to 0.889, with only 10 items above 0.50), factor loadings were also low and incoherent, with many multiple loadings (only 18 items, if not double, loaded above 0.40). We confirmed this lack of coherence between the different items by examining the correlation matrix, in which most correlations failed to exceed 0.23.

We chose to further evaluate the construct validity by means of the known-groups technique (known-groups validity). This involved employing a simple independent t-test calculated between the overall test results of the first- and third-year nursing students [43]. Test results were calculated based on a score ‘1’ for a correct answer and a score ‘0’ for a wrong answer or a ‘don’t know’ response. This resulted in a total *FAQ* score of 21.0 and 23.1 out of 36, for first- and third-year nursing students, respectively. This difference was significant (p < 0.001), supporting the construct validity of the adapted/revised *FAQ*. As shown in Table 2, 15 items (six originating from the *FAQ1* and nine originating from the *FAMHQ)* distinguish the first- and third-year students’ knowledge.

**Table 2:** **Psychometric properties of the adapted version of** ***FAQ*** **and** ***FAMHQ***

Item	Corrected item-total correlation	Alpha if item deleted	1st years students		3rd years students		Х ^2^(p-value)
			T (%)	F/DK (%)	T (%)	F/DK (%)	
(1)	.146	.721	71.2	28.8	75.4	24.6	.144
(2)	.109	.722	75.9	24.1	79.1	20.9	.244
(5)	.167	.720	79.2	20.8	84.4	15.6	.042*
(7)	.305	.712	38.8	61.3	45.9	54.1	.026*
(8)	.189	.720	38.9	61.1	44.5	55.5	.075
(9)	.275	.714	49.0	51.0	56.3	43.7	.023*
(10)	.284	.714	63.6	36.4	61.5	38.5	.517
(13)	.206	.718	76.8	23.2	80.6	19.4	.163
(16)	.258	.715	56.6	43.4	53.9	46.1	.403
(3)	.066	.724	72.8	27.2	77.3	22.7	.115
(4)	.170	.720	78.0	22.0	83.2	16.8	.046*
(6)	.051	.723	93.6	6.4	93.8	6.2	.914
(11)	.111	.722	86.1	13.9	86.7	13.3	.787
(14)	.146	.721	76.2	23.8	78.8	21.2	.339
(22)	.314	.711	25.4	74.6	28.0	72.0	.365
**(24)**	**.104**	**.723**	**79.2**	**20.8**	**75.7**	**24.3**	**.193**
*(25)*	*.289*	*.713*	*53.0*	*47.0*	*76.5*	*23.5*	.000*
*(26)*	*.164*	*.721*	*44.5*	*55.5*	*60.3*	*39.7*	.000*
*(28)*	*.148*	*.721*	*79.8*	*20.2*	*90.0*	*10.0*	.000*
*(29)*	*.207*	*.718*	*59.9*	*40.1*	*70.0*	*30.0*	.001*
*(30)*	*.175*	*.721*	*93.4*	*6.6*	*97.9*	*2.1*	.002*
*(31)*	*.322*	*.712*	*90.2*	*9.8*	*96.8*	*3.2*	.000*
(17)	.352	.708	13.6	86.4	19.2	80.8	.017*
(18)	.260	.715	6.4	93.6	8.3	91.7	.225
(19)	.225	.718	33.8	66.2	45.6	54.4	.000*
(21)	.246	.716	62.8	37.2	67.0	33.0	.180
(12)	.263	.716	75.8	24.2	74.0	26.0	.509
(15)	.325	.710	27.2	72.8	30.9	69.1	.206
*(27)*	*.245*	*.716*	*53.3*	*46.7*	*56.8*	*43.2*	*.286*
*(32)*	*.137*	*.721*	*57.9*	*42.1*	*77.6*	*22.4*	.000*
*(33)*	*.198*	*.719*	*81.8*	*18.3*	*86.1*	*13.9*	*.071*
*(34)*	*.283*	*.713*	*30.1*	*69.9*	*42.1*	*57.9*	.000*
(20)	.274	.715	10.6	89.4	12.4	87.6	.400
**(23)**	**.056**	**.724**	**89.3**	**10.8**	**92.4**	**7.6**	**.107**
*(35)*	*.308*	*.712*	*45.3*	*54.7*	*55.9*	*44.1*	.001*
*(36)*	*.301*	*.712*	*38.3*	*61.7*	*42.1*	*57.9*	*.241*

T = true. F = false. DK = don’t know. *= statistically significant with p < .05.

Regular font = items originating from the Facts on Aging Quiz (FAQ) version 1.

Bold font = items were added (as recommended by the Flemish expert panel).

*Italicized font* = items originating from the Facts on Aging and Mental Health Quiz (FAMHQ).

Internal consistency of the adapted *FAQ* instrument was evaluated by calculating Cronbach’s alpha scores. This resulted in an acceptable alpha coefficient of 0.723 for the revised scale. Additionally, alpha-if-item-deleted and item-to-total correlations were calculated (See Table 2). Deletion of an item produced minimal changes to the alpha coefficient, ranging from 0.708 and 0.724. However, item-to-total correlation ranged from 0.051 to 0.352 (mean = 0.213). Although some items had a very low item-to-total correlation—i.e., 0.066 (item 3), 0.051 (item 6), 0.056 (item 23)—we decided to retain these items, as they are all related to physical decline, an important factor of the ageing process [49].

## Discussion

In this study, we aimed to create a revised version of the *FAQ* that would better evaluate nursing students’ knowledge about ageing and care for older persons. The new instrument is based on the *FAQ1* and *FAMHQ,* originally developed by Palmore [32–34] and two additional items suggested by the expert panel. Development of the revised instrument proceeded in two phases: (1) translation, adaptation, and validation; and (2) evaluation of the revised instrument’s construct validity and internal consistency. The first phase produced a provisional instrument with 22 items from the original *FAQ1,* two *de novo* items about life expectancy and risk of physical injury, and 12 items from the original *FAMHQ.* The second phase was based on the results of an exploratory study in which the revised instrument was administered to 1141 first- and third-year nursing students in colleges in Flanders, Belgium. The final version of the revised and extended *FAQ* scale consisted of 36 items, which are answered with ‘true’, ‘false’, or ‘don’t know’. An external expert panel of educators confirmed the face validity of the revised instrument, and evaluation of its internal consistency produced a Cronbach’s alpha of 0.723. Explorative factor analysis failed to produce a simple structure. However, using the known-groups technique, we confirmed the construct validity of the revised instrument.

As expected, explorative factor analysis failed to reveal different underlying constructs. Inclusion of various factors produced a substantively incoherent model, and factor loadings were generally low. On the basis of these results and on other results reported in the literature on the factor structure of *Palmore’s FAQs* [42, 44], we surmise that the overall underlying concept could, therefore, be related more to general knowledge about older persons, ageing, and gerontological care. This suggested underlying concept is very broad and covers many different aspects. However, we did confirm construct validity of our revised version of the *FAQ* in a large sample of nursing students using the known-groups technique. With this approach, the new instrument was able to discriminate (p < 0.001) between the knowledge of first- and third-year nursing students about older persons and ageing.

Another finding regarding the new instrument’s discriminatory power was that almost all items that were incorporated from the *FAMHQ*, except three (items 3, 29, and 33)*,* significantly discriminated between first- and third-year nursing students’ knowledge. By contrast, only 6 of 24 items incorporated from the *FAQ1* showed similar performance. This difference might indicate that overall knowledge about older people, ageing, and gerontological care tapped into by the *FAQ1* items is more or less the same for first- and third-year nursing students. By extension, knowledge about the cognitive and mental state of older persons tapped into by the *FAMHQ* items is likely knowledge that is gained through internship experiences and more extensive training packages that students receive in the second and third year of nursing education.

Overall knowledge about older persons and gerontological care is a difficult construct to measure and operationalize in a measurement instrument. Palmore’s research provides a useful starting point, a valuable framework in this area on which to build. Our study demonstrates the overall utility and value of Palmore’s work, despite the necessity of some adjustments for its use in Belgium, Flanders. This fact is also reflected by the numerous translations and adaptations for use in different regions [22, 27, 29, 31, 45, 50–55]. Nevertheless, we must also note a caveat about its unrestricted use. In line with previous research on the use of *Palmore’s FAQs*, we agree that it is primarily suitable in non-research–based settings or in discussion groups [42–45, 47]. This is true also for the revised (Flemish) version.

### Limitations

Some limitations of the current study should be discussed. First, no quantitative method was used to evaluate face and content validity. The latter were only supported by oral consensus among all experts. The internal consistency of the scale reached 0.723, which is a reasonable alpha coefficient, but not an excellent one, given the number of items included in the instrument [48]. Also in the current study, the ‘don’t know’ response was added, because this format reduces guessing, thereby improving the reliability of both scales [47]. This would mean an increase in Cronbach’s alpha. However, the Cronbach’s alpha of the items of the *FAQ1* (α = 0.65) reported in this study is comparable to those in previous studies; i.e., varying from 0.45 to 0.66 [43–45].

Although the forward translation was conducted by four content experts with profound knowledge of the English language, due to time and financial constraints no formal backward translation of the revised FAQ was conducted. Backwards translation is still considered as a necessary component in instrument development, and its lack should be considered as a limitation [56].

On the basis of reasonable reliability and validity demonstrated for our revised *FAQ,* we decided against making any ‘last minute’ changes (e.g., deleting some of the items with a low item-to-total correlation), as these items were all agreed upon as being relevant by the expert panel. However, we recommend that, in the future, researchers’ focus should be on further evaluating the psychometric properties of the revised *FAQ* scale (or other revisions of *Palmore’s FAQs)*. The results of our study also strongly suggest a revision of a number of items, if the intention is to use it for scientific research. For example, double-formulation should be implemented for some items, and any underlying attitude bias should be eliminated for a number of items. Using the instrument in different settings and different populations should be evaluated, as stability of the revised instrument might be questioned with only the present testing data.

## Conclusion

*Palmore’s FAQs* were originally developed to encourage healthcare workers to participate in group discussion about older persons and gerontological care. On the basis of the findings of this study, we conclude that our revised and extended version of the *FAQ* can also be used to evaluate nursing students’ knowledge about older persons and gerontological care in Flanders, Belgium, as reasonable reliability and validity were established. Future research, however, is needed to further evaluate and confirm the psychometric properties of this scale in different groups of students and in different settings.

## Electronic supplementary material

Additional file 1: **Translation and adaptation of the Fact’s on aging Quiz 1 (FAQ1) into Dutch.** (DOCX 16 KB)

Additional file 2: **Translation and adaptation of the Fact’s on aging and mental health Quiz (FAMHQ) into Dutch.** (DOCX 16 KB)

Additional file 3: **Revised and extended version of** ***FAQ1*** **and** ***FAMHQ*** **(in Dutch).** (DOCX 17 KB)
