# Supplementary material for: Internal consistency and construct validity assessment of a revised Facts on Aging Quiz for Flemish nursing students: an exploratory study
Source: BMC Geriatr. 2014 Dec 3;14:128. doi: 10.1186/1471-2318-14-128 (PMC4267412; doi:10.1186/1471-2318-14-128)
Supplement: Supplementary file 1 — Additional file 1: Translation and adaptation of the Fact’s on aging Quiz 1 (FAQ1) into Dutch. (DOCX 16 KB) [file 12877_2014_1063_MOESM1_ESM.docx]

**Appendix 1: Translation and adaptation of the Fact’s on aging Quiz 1 (FAQ1) into Dutch.**

| **Fact’s on aging Quiz 1 (FAQ1)**  **(Palmore et al. 2005)** | **Flemish version** |
| --- | --- |
| The majority of old people (age 65 or older) are senile (have defective memory, are disoriented, or demented). | De meerderheid van de ouderen (65 jaar of ouder) heeft geheugenproblemen, is gedesoriënteerd, of lijdt aan dementie. |
| The five senses (sight, hearing, taste, touch, and smell) all weaken in old age. | Bij het ouder worden gaan alle vijf zintuigen (zicht, gehoor, smaak, gevoel en geur) erop achteruit. |
| The majority of old people have no interest in, or capacity for, sexual relations. | De meerderheid van de ouderen heeft geen interesse in, noch de capaciteit tot seksuele relaties. |
| Lung vital capacity tends to decline in old age. | De vitale longcapaciteit neemt geleidelijk aan af bij het ouder worden. |
| The majority of old people feel miserable most of the time. | De meerderheid van de ouderen voelt zich meestal ellendig. |
| Physical strength tends to decline in old age. | Fysieke kracht neemt af bij het ouder worden. |
| At least 10% of the aged are living in long-stay institutions (such as nursing homes, mental hospitals, homes for the aged, etc.). | Meer dan één op de vier van de 65-plussers leeft in een chronische zorgsetting (zoals rusthuizen,woonzorgcentra, psychiatrische instellingen). |
| Aged drivers have fewer accidents per driver than those under the age 65. | **not included** |
| Older workers usually cannot work as effectively as younger workers. | Oudere mensen werken niet zo productief als jongere mensen. |
| Over 75% of the aged are healthy enough to do their normal activities without help. | De meerderheid van de ouderen zijn gezond genoeg om hun normale activiteiten uit te voeren zonder hulp. |
| The majority of old people are unable to adapt to change. | De meerderheid van de ouderen kan zich niet aanpassen aan verandering. |
| Old people usually take longer to learn something new. | Ouderen hebben meer tijd nodig om iets nieuws aan te leren. |
| Depression is more frequent among the elderly than among younger people. | Depressie komt weinig voor bij oudere mensen. |
| Older people tent to react more slowly than younger people do. | Oudere mensen hebben de neiging om langzamer te reageren dan jongere mensen. |
| In general, old people tend to be pretty much alike. | Algemeen kunnen we stellen dat ouderen sterk op elkaar lijken. |
| The majority of old people say they are seldom bored. | De meerderheid van de ouderen zegt dat ze zich zelden vervelen. |
| The majority of old people are socially isolated. | De meerderheid van de ouderen is sociaal geïsoleerd. |
| Older workers have fewer accidents than younger workers do. | Oudere mensen die werken, hebben minder ongelukken dan jongere mensen die werken. |
| Over 20% of the population are now age 65 or over. | Op dit moment is in Vlaanderen meer dan 30% van de populatie 65 jaar of ouder. |
| The majority of medical practitioners give low priority to the aged. | De meerderheid van de gezondheidswerkers geeft een lage prioriteit aan het werken en het zorgen voor ouderen. |
| The majority of old people have incomes below the poverty line (as defined by the federal government). | De meerderheid van de ouderen heeft een inkomen onder het bestaansminimum (tussen €700/maand en €900/maand) |
| The majority of old people are working or would like to have some kind of work to do (including housework and volunteer work). | De meerderheid van de ouderen is aan het werk of zou graag één of ander werk uitvoeren (het huishouden,vrijwilligerswerk, zorg voor kleinkinderen etc. inbegrepen) |
| Older people tend to become more religious as they age. | **not included** |
| The majority of old people say they are seldom irritated or angry. | De meerderheid van de ouderen zegt zelden geïrriteerd of boos te zijn. |
| The health and economic status of old people will be about the same or worse in the year 2010 (compared to younger people). | **not included** |
| © Erdman Palmore: reproduced and translated with kind permission of Prof. Dr. Em. Erdman Palmore | |
