# Supplementary material for: Internal consistency and construct validity assessment of a revised Facts on Aging Quiz for Flemish nursing students: an exploratory study
Source: BMC Geriatr. 2014 Dec 3;14:128. doi: 10.1186/1471-2318-14-128 (PMC4267412; doi:10.1186/1471-2318-14-128)
Supplement: Supplementary file 2 — Additional file 2: Translation and adaptation of the Fact’s on aging and mental health Quiz (FAMHQ) into Dutch. (DOCX 16 KB) [file 12877_2014_1063_MOESM2_ESM.docx]

**Appendix 2: Translation and adaptation of the Fact’s on aging and mental health Quiz (FAMHQ) into Dutch.**

| **Fact’s on aging and mental health Quiz (FAMHQ)**  **(Palmore 1998)** | **Flemish version** |
| --- | --- |
| The majority of persons over 65 have some mental illness severe enough to impair their abilities. | De meerderheid van de personen boven 65 jaar heeft een psychische ziekte die in die mate ernstig is dat het hun normale functioneren beperkt. |
| Cognitive impairment (memory loss, disorientation, or confusion) is an inevitable part of the aging process. | Een cognitieve stoornis (geheugenverlies, desoriëntatie of verwardheid) is een onvermijdelijk onderdeel van het verouderingsproces. |
| If an older mental patient makes up false stories, it is best to point out that he or she is lying. | **not included** |
| The prevalence of neurosis and schizophrenia increases in old age. | **not included** |
| Suicide rates increase with age for women past 45. | **not included** |
| Suicide rate increase with age for men past 45. | **not included** |
| Fewer of the aged have mental impairments, when all types are added together, than other age groups. | **not included** |
| The primary mental illness of the elderly is cognitive impairment. | **not included** |
| Alzheimer’s disease (progressive senile dementia) is the most common type of chronic cognitive impairment among the aged. | De ziekte van Alzheimer is het meest voorkomende type van chronische cognitieve stoornissen bij ouderen. |
| There is no cure for Alzheimer’s disease. | Er is geen behandeling (die tot genezing leidt) bij de ziekte van Alzheimer. |
| Most patients with Alzheimer’s disease act the same way. | De meeste patiënten met de ziekte van Alzheimer gedragen zich op dezelfde manier. |
| Organic brain impairment is easy to distinguish from functional mental illness. | **not included** |
| It is best not to look directly at older mental patients when you are talking to them. | **not included** |
| It is best to avoid talking to demented patients, because it may increase their confusion. | Je vermijdt beter te praten met dementerende ouderen, omdat dit hun verwardheid kan doen toenemen. |
| Demented patients should not be allowed to talk about their past because it may depress them. | Dementerende patiënten mogen niet worden toegelaten te praten over hun verleden, omdat het hen depressief kan maken. |
| The prevalence of cognitive impairment increases in old age. | De prevalentie van cognitieve stoornissen neemt toe met de leeftijd. |
| Isolation and hearing loss are the most frequent causes of paranoid disorders in old age. | **not included** |
| Poor nutrition may produce mental illness among the elderly. | **not included** |
| Mental illness is more prevalent among the elderly with less income and education. | Psychische ziekten komen vaker voor (hebben een hogere prevalentie) bij ouderen met een lager inkomen en een lagere opleiding. |
| The majority of nursing home patients suffer from mental illness. | De meerderheid van de patiënten in een woonzorgcentrum (rusthuis, ROB, RVT) lijdt aan een psychische ziekte. |
| The elderly have fewer sleep problems than younger persons. | Oudere personen hebben minder slaapproblemen dan jongere personen. |
| Major depression is more prevalent among the elderly than among younger persons. | Ernstige (majeure) depressie komt vaker voor bij oudere dan bij jongere personen. |
| Widowhood is more stressful for older than for younger women. | **not included** |
| More of the aged use mental health services than do younger persons. | **not included** |
| Psychotherapy is usually ineffective with older patients. | **not included** |

© Erdman Palmore: reproduced and translated with kind permission of Prof. Dr. Em. Erdman Palmore
