# Supplementary material for: Internal consistency and construct validity assessment of a revised Facts on Aging Quiz for Flemish nursing students: an exploratory study
Source: BMC Geriatr. 2014 Dec 3;14:128. doi: 10.1186/1471-2318-14-128 (PMC4267412; doi:10.1186/1471-2318-14-128)
Supplement: Supplementary file 3 — Additional file 3: Revised and extended version of FAQ1 and FAMHQ (in Dutch). (DOCX 17 KB) [file 12877_2014_1063_MOESM3_ESM.docx]

**Appendix 3. Revised and extended version of *FAQ1* and *FAMHQ* (in Dutch)**

| Number | Item |
| --- | --- |
| (1) | De meerderheid van de ouderen (65 jaar of ouder) heeft geheugenproblemen, is gedesoriënteerd, of lijdt aan dementie. |
| (2) | De meerderheid van de ouderen heeft geen interesse in, noch de capaciteit tot seksuele relaties. |
| (3) | Bij het ouder worden gaan alle vijf zintuigen (zicht, gehoor, smaak, gevoel en geur) erop achteruit. |
| (4) | De vitale longcapaciteit neemt geleidelijk aan af bij het ouder worden. |
| (5) | De meerderheid van de ouderen voelt zich meestal ellendig. |
| (6) | Fysieke kracht neemt af bij het ouder worden. |
| (7) | Meer dan 25% van de 65-plussers leeft in een chronische zorgsetting (zoals rusthuizen, woonzorgcentra, psychiatrische instellingen). |
| (8) | Oudere mensen werken niet zo productief als jongere mensen. |
| (9) | De meerderheid van de ouderen kan zich niet aanpassen aan verandering. |
| (10) | De meerderheid van de ouderen zijn gezond genoeg om hun normale activiteiten uit te voeren zonder hulp. |
| (11) | Ouderen hebben meer tijd nodig om iets nieuws aan te leren. |
| (12) | Depressie komt weinig voor bij oudere mensen. |
| (13) | Algemeen kunnen we stellen dat ouderen sterk op elkaar lijken. |
| (14) | Oudere mensen hebben de neiging om langzamer te reageren dan jongere mensen. |
| (15) | De meerderheid van de ouderen zegt dat ze zich zelden vervelen. |
| (16) | De meerderheid van de ouderen is sociaal geïsoleerd. |
| (17) | Oudere mensen die werken, hebben minder ongelukken dan jongere mensen die werken. |
| (18) | Op dit moment is in Vlaanderen meer dan 30% van de populatie 65 jaar of ouder. |
| (19) | De meerderheid van de gezondheidswerkers geeft een lage prioriteit aan het werken en het zorgen voor ouderen. |
| (20) | De meerderheid van de ouderen heeft een inkomen onder het bestaansminimum (tussen €700/maand en €900/maand) |
| (21) | De meerderheid van de ouderen is aan het werk of zou graag één of ander werk uitvoeren (het huishouden, vrijwilligerswerk, zorg voor kleinkinderen etc. inbegrepen) |
| (22) | De meerderheid van de ouderen zegt zelden geïrriteerd of boos te zijn. |
| **(23)** | **Het risico op fysiek letsel (bv. gebroken heup en/of pols) is even groot bij oudere als bij jongere mensen.** |
| **(24)** | **De levensverwachting op 65 jaar is ongeveer dezelfde voor de man als voor de vrouw.** |
| *(25)* | *De meerderheid van de personen boven 65 jaar heeft een psychische ziekte die in die mate ernstig is dat het hun normale functioneren beperkt.* |
| *(26)* | *Een cognitieve stoornis (geheugenverlies, desoriëntatie of verwardheid) is een onvermijdelijk onderdeel van het verouderingsproces.* |
| *(27)* | *De ziekte van Alzheimer is het meest voorkomende type van chronische cognitieve stoornissen bij ouderen.* |
| *(28)* | *Er is geen behandeling (die tot genezing leidt) bij de ziekte van Alzheimer.* |
| *(29)* | *De meeste patiënten met de ziekte van Alzheimer gedragen zich op dezelfde manier.* |
| *(30)* | *Je vermijdt beter te praten met dementerende ouderen, omdat dit hun verwardheid kan doen toenemen.* |
| *(31)* | *Dementerende patiënten mogen niet worden toegelaten te praten over hun verleden, omdat het hen depressief kan maken.* |
| *(32)* | *De prevalentie van cognitieve stoornissen neemt toe met de leeftijd.* |
| *(33)* | *Oudere personen hebben minder slaapproblemen dan jongere personen.* |
| *(34)* | *Psychische ziekten komen vaker voor (hebben een hogere prevalentie) bij ouderen met een lager inkomen en een lagere opleiding.* |
| *(35)* | *De meerderheid van de patiënten in een woonzorgcentrum (rusthuis, ROB, RVT) lijdt aan een psychische ziekte.* |
| *(36)* | *Ernstige (majeure) depressie komt vaker voor bij oudere dan bij jongere personen.* |

Normaalgedrukt= items overgenomen vanuit de Facts on Aging Quiz version 1.

**Vetgedrukt** = items werden toegevoegd (op aanraden van het Vlaamse expertenpanel).

*Cursief gedrukt* = items overgenomen vanuit de Facts on Aging and Mental Health Quiz.
